# Supplementary material for: Rational Design of Lipase ROL to Increase Its Thermostability for Production of Structured Tags
Source: Int J Mol Sci. 2022 Aug 23;23(17):9515. doi: 10.3390/ijms23179515 (PMC9455606; doi:10.3390/ijms23179515)
Supplement: Supplementary file 1 [file ijms-23-09515-s001.zip › ijms-1865493-supplementary.pdf]

MVPVSGKSGSSTTAVSASDNSALPPLISSRCAPPSNKGSKSDLQAEPYYMQKNTEWYESHGGNLTSI
GKRDDNLVGGMTLDLPSDAPPISLSGSTNSASDGGKVAAATTAQIQEFTKYAGIAATAYCRSVVPG
NKWDCVQCQKWVPDGKIITTFTSLLSDTNGYVLRSDKQKTIYLVFRGTNSFRSAITDIVFNFSDYKPV
KGAKVHAGFLSSYEQVVNDYFPVIQEQLTANPTYKVIVTGHSLGGAQALLAGMDLYQREPRLSPK
NLSIFTVGGPRVGNPTFAYYVESTGIPFQRTVHKRDIVPHVPPQSFGFLHPGVESWIKSGTSNVQICTS
EIETKDCSNSIVPFTSLLDHLSYFDINEGSCL

Figure S1. Sequence of wild-type ROL.

CTTTGTAGCCTTAGACATGACTGTTCCTCAGTTCAAGTTGGGCACTTACGAGAAGACCGGTCTTGCTAGA
TTCTAATCAAGAGGATGTCAGAATGCCATTTGCCTGAGAGATGCAGGCTTCATTTTTGATACTTTTTTATT
TGTAACCTATATAGTATAGGATTTTTTTTGTCATTTTGTTTCTTCTCGTACGAGCTTGCTCCTGATCAGCCT
ATCTCGCAGCTGATGAATATCTTGTGGTAGGGGTTTGGGAAAATCATTCGAGTTTGATGTTTTTCTTGGTA
TTTCCCACTCCTCTTCAGAGTACAGAAGATTAAGTGAGACTTTCGTTTGTGCGGATCCGCTGGGATCCTA
ATGCGGTAGTTTATCACAGTTAAATTGCTAACGCAGTCAGGCACCGTGTATGAAATCTAACAATGCGCTC
ATCGTCATCCTCGGCACCGTCACCCTGGATGCTGTAGGCATAGGCTTGGTTATGCCGGTACTGCCGGGCC
TCTTGCGGGATATCGTCCATTCCGACAGCATCGCCAGTCACTATGGCGTGCTGCTAGCGCTATATGCGTT
GATGCAATTTCTATGCGCACCCGTTCTCGGAGCACTGTCCGACCGCTTTGGCCGCCGCCCAGTCCTGCTC
GCTTCGCTACTTGGAGCCACTATCGACTACGCGATCATGGCGACCACACCCGTCCTGTGGATCTATCGAA
TCTAAATGTAAGTTAAAATCTCTAAATAATTAAATAAGTCCCAGTTTCTCCATACGAACCTTAACAGCAT
TGCGGTGAGCATCTAGACCTTCAACAGCAGCCAGATCCATCACTGCTTGGCCAATATGTTTCAGTCCCTC
AGGAGTTACGTCTTGTGAAGTGATGAACTTCTGGAAGGTTGCAGTGTTAACTCCGCTGTATTGACGGGCA
TATCCGTACGTTGGCAAAGTGTGGTTGGTACCGGAGGAGTAATCTCCACAACTCTCTGGAGAGTAGGCA
CCAACAAACACAGATCCAGCGTGTTGTACTTGATCAACATAAGAAGAAGCATTCTCGATTTGCAGGATC
AAGTGTTCAGGAGCGTACTGATTGGACATTTCCAAAGCCTGCTCGTAGGTTGCAACCGATAGGGTTGTAG
AGTGTGCAATACACTTGCGTACAATTTCAACCCTTGGCAACTGCACAGCTTGGTTGTGAACAGCATCTTC
AATTCTGGCAAGCTCCTTGTCTGTCATATCGACAGCCAACAGAATCACCTGGGAATCAATACCATGTTCA
GCTTGAGACAGAAGGTCTGAGGCAACGAAATCTGGATCAGCGTATTTATCAGCAATAACTAGAACTTCA
GAAGGCCCAGCAGGCATGTCAATACTACACAGGGCTGATGTGTCATTTTGAACCATCATCTTGGCAGCA
GTAACGAACTGGTTTCCTGGACCAAATATTTTGTCACACTTAGGAACAGTTTCTGTTCCGTAAGCCATAG
CAGCTACTGCCTGGGCGCCTCCTGCTAGCACGATACACTTAGCACCAACCTTGTGGGCAACGTAGATGA
CTTCTGGGGTAAGGGTACCATCCTTCTTAGGTGGAGATGCAAAAACAATTTCTTTGCAACCAGCAACTTT
GGCAGGAACACCCAGCATCAGGGAAGTGGAAGGCAGAATTGCGGTTCCACCAGGAATATAGAGGCCA
ACTTTCTCAATAGGTCTTGCAAAACGAGAGCAGACTACACCAGGGCAAGTCTCAACTTGCAAAGTCTCC
GTTAGTTGAGCTTCATGGAATTTCCTGACGTTATCTATAGAGAGATCAATGGCTCTCTTAACGTTATCTGG
CAATTGCATAAGTTCCTCTGGGAAAGGAGCTTCTAACACAGGTGTCTTCAAAGCGACTCCATCAAACTTG
GCAGTTAGTTCTAAAAGGGCTTTGTCACCATTTTGACGAACATTGTCGACAATTGGTTTGACTAATTCCAT
AATCTGTTCCGTTTTCTGGATAGGACGACGAAGGGCATCTTCAATTTCTTGTGAGGAGGCCTTAGAAACG
TCAATTTTGCACAATTCAATACGACCTTCAGAAGGGACTTCTTTAGGTTTGGATTCTTCTTTAGGTTGTTCC
TTGGTGTATCCTGGCTTGGCATCTCCTTTCCTTCTAGTGACCTTTAGGGACTTCATATCCAGGTTTCTCTCC
ACCTCGTCCAACGTCACACCGTACTTGGCACATCTAACTAATGCAAAATAAAATAAGTCAGCACATTCC
CAGGCTATATCTTCCTTGGATTTAGCCTCGGCCAACTCGTCGGCCTCCTCCCTAATTTTAGCGTTCAACAA
AACTTCGTCGTCAAATAACCGTTTGGTATAAGAACCTTCTGGAGCATTGCTCTTACGATCCCACAAGGTG
GCTTCCATGGCTCTAAGACCCTTTGATTGGCCAAAACAGGAAGTGCGTTCCAAGTGACAGAAACCAACA
CCTGTTTGTTCAACCACAAATTTCAAGCAGTCTCCATCACAATCCAATTCGATACCCAGCAACTTTTGAG
TTGCTCCAGATGTAGCACCTTTATACCACAAACCGTGACGACGAGATTGGTAGACTCCAGTTTGTGTCCT
TATAGCCTCCGGAATAGACTTTTTGGACGAGTACACCAGGCCCAACGAGTAATTAGAAGAGTCAGCCAC
CAAAGTAGTGAATAGACCATCGGGGCGGTCAGTAGTCAAAGACGCCAACAAAATTTCACTGACAGGGA
ACTTTTTGACATCTTCAGAAAGTTCGTATTCAGTAGTCAATTGCCGAGCATCAATAATGGGGATTATACC
AGAAGCAACAGTGGAAGTCACATCTACCAACTTTGCAGTCTCAGAAAAAGCATAAACAGTTCTACTACC
GCCATTAGTGAAACTTTTCAAATCGCCCAGTGGAGAAGAAAAAGGCACAGCGATACTAGCATTAGCGG
GCAAGGATGCAACTTTATCAACCAGGGTCCTATAGATAACCCTAGCGCCTGGGATCATCCTTTGGACAA
CTCTTTCTGCCAAATCTAGGTCCAAAATCACTTCATTGATACCATTATTGTACAACTTGAGCAAGTTGTCG
ATCAGCTCCTCAAATTGGTCCTCTGTAACGGATGACTCAACTTGCACATTAACTTGAAGCTCAGTCGATT
GAGTGAACTTGATCAGGTTGTGCAGCTGGTCAGCAGCATAGGGAAACACGGCTTTTCCTACCAAACTCA
AGGAATTATCAAACTCTGCAACACTTGCGTATGCAGGTAGCAAGGGAAATGTCATACTTGAAGTCGGAC
AGTGAGTGTAGTCTTGAGAAATTCTGAAGCCGTATTTTTATTATCAGTGAGTCAGTCATCAGGAGATCCT
CTACGCCGGACGCATCGTGGCCGGCATCACCGGCGCCACAGGTGCGGTTGCTGGCGCCTATATCGCCGA
CATCACCGATGGGGAAGATCGGGCTCGCCACTTCGGGCTCATGAGCGCTTGTTTCGGCGTGGGTATGGTG
GCAGGCCCCGTGGCCGGGGGACTGTTGGGCGCCATCTCCTTGCATGCACCATTCCGGAGACTAACCCAC
ACACCATAGCTTCAAAATGTTTCTACTCCTTTTTTACTCTTCCAGATTTTCTCGGACTCCGCGCATCGCCGT
ACCACTTCAAAACACCCAAGCACAGCATACTAAATTTCCCCTCTTTCTTCCTCTAGGGTGTCGTTAATTAC
CCGTACTAAAGGTTTGGAAAAGAAAAAAAAGACCGCCTCGTTTCTTTTTCTTCGTCGAAAAAGGCAATA
AAAATTTTTATCACGTTTCTTTTTCTTGAAAATTTTTTTTTTTGATTTTTTTCTCTTTCGATGACCTCCCATTG
ATATTTAAGTTAATAAACGGTCTTCAATTTCTCAAGTTTCAGTTTCATTTTTCTTGTTCTATTACAACTTTTT
TTACTTCTTGCTCATTAGAAAGAAAGCATAGCAATCTAATCTAAGGGGCGGTGTTGACAATTAATCATCG

GCATAGTATATCGGCATAGTATAATACGACAAGGTGAGGAACTAAACCATGGCCAAGTTGACCAGTGC
CGTTCCGGTGCTCACCGCGCGCGACGTCGCCGGAGCGGTCGAGTTCTGGACCGACCGGCTCGGGTTCTC
CCGGGACTTCGTGGAGGACGACTTCGCCGGTGTGGTCCGGGACGACGTGACCCTGTTCATCAGCGCGGT
CCAGGACCAGGTGGTGCCGGACAACACCCTGGCCTGGGTGTGGGTGCGCGGCCTGGACGAGCTGTACG
CCGAGTGGTCGGAGGTCGTGTCCACGAACTTCCGGGACGCCTCCGGGCCGGCCATGACCGAGATCGGCG
AGCAGCCGTGGGGGCGGGAGTTCGCCCTGCGCGACCCGGCCGGCAACTGCGTGCACTTCGTGGCCGAG
GAGCAGGACTGACACGTCCGACGGCGGCCCACGGGTCCCAGGCCTCGGAGATCCGTCCCCCTTTTCCTT
TGTCGATATCATGTAATTAGTTATGTCACGCTTACATTCACGCCCTCCCCCCACATCCGCTCTAACCGAAA
AGGAAGGAGTTAGACAACCTGAAGTCTAGGTCCCTATTTATTTTTTTATAGTTATGTTAGTATTAAGAAC
GTTATTTATATTTCAAATTTTTCTTTTTTTTCTGTACAGACGCGTGTACGCATGTAACATTATACTGAAAAC
CTTGCTTGAGAAGGTTTTGGGACGCTCGAAGGCTTTAATTTGCAAGCTGGAGACTAACATGTGAGCAAA
AGGCCAGCAAAAGGCCAGGAACCGTAAAAAGGCCGCGTTGCTGGCGTTTTTCCATAGGCTCCGCCCCCC
TGACGAGCATCACAAAAATCGACGCTCAAGTCAGAGGTGGCGAAACCCGACAGGACTATAAAGATACC
AGGCGTTTCCCCCTGGAAGCTCCCTCGTGCGCTCTCCTGTTCCGACCCTGCCGCTTACCGGATACCTGTCC
GCCTTTCTCCCTTCGGGAAGCGTGGCGCTTTCTCAATGCTCACGCTGTAGGTATCTCAGTTCGGTGTAGGT
CGTTCGCTCCAAGCTGGGCTGTGTGCACGAACCCCCCGTTCAGCCCGACCGCTGCGCCTTATCCGGTAAC
TATCGTCTTGAGTCCAACCCGGTAAGACACGACTTATCGCCACTGGCAGCAGCCACTGGTAACAGGATT
AGCAGAGCGAGGTATGTAGGCGGTGCTACAGAGTTCTTGAAGTGGTGGCCTAACTACGGCTACACTAGA
AGGACAGTATTTGGTATCTGCGCTCTGCTGAAGCCAGTTACCTTCGGAAAAAGAGTTGGTAGCTCTTGAT
CCGGCAAACAAACCACCGCTGGTAGCGGTGGTTTTTTTGTTTGCAAGCAGCAGATTACGCGCAGAAAAA
AAGGATCTCAAGAAGATCCTTTGATCTTTTCTACGGGAACGTTTTTGTAGAAATGTCTTGGTGTCCTCGTC
CAATCAGGTAGCCATCTCTGAAATATCTGGCTCCGTTGCAACTCCGAACGACCTGCTGGCAACGTAAAA
TTCTCCGGGGTAAAACTTAAATGTGGAGTAATGGAACCAGAAACGTGTCTTCCCTTCTCTCTCCTTCCAC
CGCCCGTTACCGTCCCTAGGAAATTTTACTCTGCTGGAGAGCTTCTTCTACGGCCCCCTTGCAGCAATGCT
CTTCCCAGCATTACGTTGCGGGTAAAACGGAGGTCGTGTACCCGACCTAGCAGCCCAGGGATGGAAAAG
TCCCGGCCGTCGCTGGCAATAATAGCGGGCGGACGCATGTCATGAGATTATTGGAAACCACCAGAATCG
AATATAAAAGGCGAACACCTTTCCCAATTTTGGTTTCTCCTGACCCAAAGACTTTAAATTTAATTTATTTG
TCCCTATTTCAATCAATTGAACAACTATCTATGAGATTTCCTTCAATTTTTACTGCTGTTTTATTCGCAGCA
TCCTCCGCATTAGCTGCTCCAGTCAACACTACAACAGAAGATGAAACGGCACAAATTCCGGCTGAAGCT
GTCATCGGTTACTCAGATTTAGAAGGGGATTTCGATGTTGCTGTTTTGCCATTTTCCAACAGCACAAATA
ACGGGTTATTGTTTATAAATACTACTATTGCCAGCATTGCTGCTAAAGAAGAAGGGGTATCTCTCGAGAA
AAGAGAGGCTGAAGCTGTGATGGTTCCAGTTTCTGGTAAGTCTGGTTCCTCCACTACTGCTGTTTCTGCTT
CTGACAACTCTGCCTTGCCACCATTGATCTCTTCTAGATGTGCTCCACCATCCAACAAGGGTTCTAAGTCT
GACTTGCAAGCCGAGCCTTACTACATGCAAAAGAACACTGAATGGTACGAGTCCCACGGTGGTAACTTG
ACTTCCATCGGTAAGAGAGATGACAACCTGGTTGGTGGTATGACCTTGGATTTGCCATCTGACGCTCCTC
CAATTTCTTTGTCCGGTTCTACCAACTCTGCTTCCGATGGTGGTAAAGTTGCTGCTGCTACTACTGCTCAG
ATCCAAGAGTTCACTAAGTACGCTGGTATCGCTGCTACTGCCTACTGTAGATCTGTTGTCCCAGGTAACA
AGTGGGACTGTGTTCAGTGTCAAAAGTGGGTTCCAGACGGTAAGATTATCACCACCTTCACTTCCCTGTT
GTCCGACACTAACGGTTACGTTTTGAGATCCGACAAGCAAAAGACCATCTACCTGGTGTTCAGAGGAAC
TAACTCCTTCAGATCCGCTATCACCGACATCGTGTTCAACTTCTCTGACTACAAGCCAGTCAAGGGTGCT
AAGGTTCACGCTGGTTTTTTGTCCTCTTACGAGCAGGTTGTGAACGACTACTTCCCAGTTATCCAAGAGC
AGTTGACTGCTTATCCAACCTACAAGGTTATCGTTACCGGTCACTCTCTTGGTGGTGCTCAAGCTTTGTTG
GCTGGTATGGACTTGTACCAGAGAGAGCCAAGATTGTCCCCAAAGAACTTGTCCATCTACACCGTTGGT
GGTCCAAGAGTCGGTAACCCAACTTTCGCTTACTACGTTTGCTCCACTGGTATCCCATTCCATAGAACCG
TTCACAAGAGAGACATCGTTCCACACTTGCCACCTCAATCCTTTGGTTTCTTGCACCCAGGTGTTGAGTCC
TGGATTAAGTCCGGTACTTCCAACGTTCAGATCTGCACTTCCTGCATCGAGACTAAGGACTGTTCCAACT
CTATCGTCCCTTTCACTTCTTTGCTGGACCACTTGTCCTACTTCGGTATTAACGAGGGTTTGTGTTTGTAG

Figure S2. Vector sequence of pFAi2-GAPp-ROL.

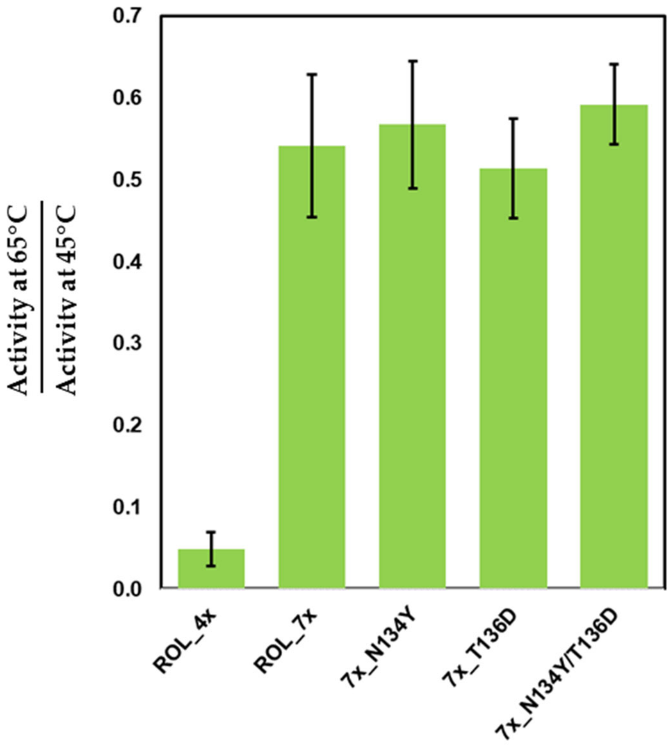

Figure S3. DTNB assay to compare the thermostability of ROL_4x, ROL_7x and ROL_7x mutants.

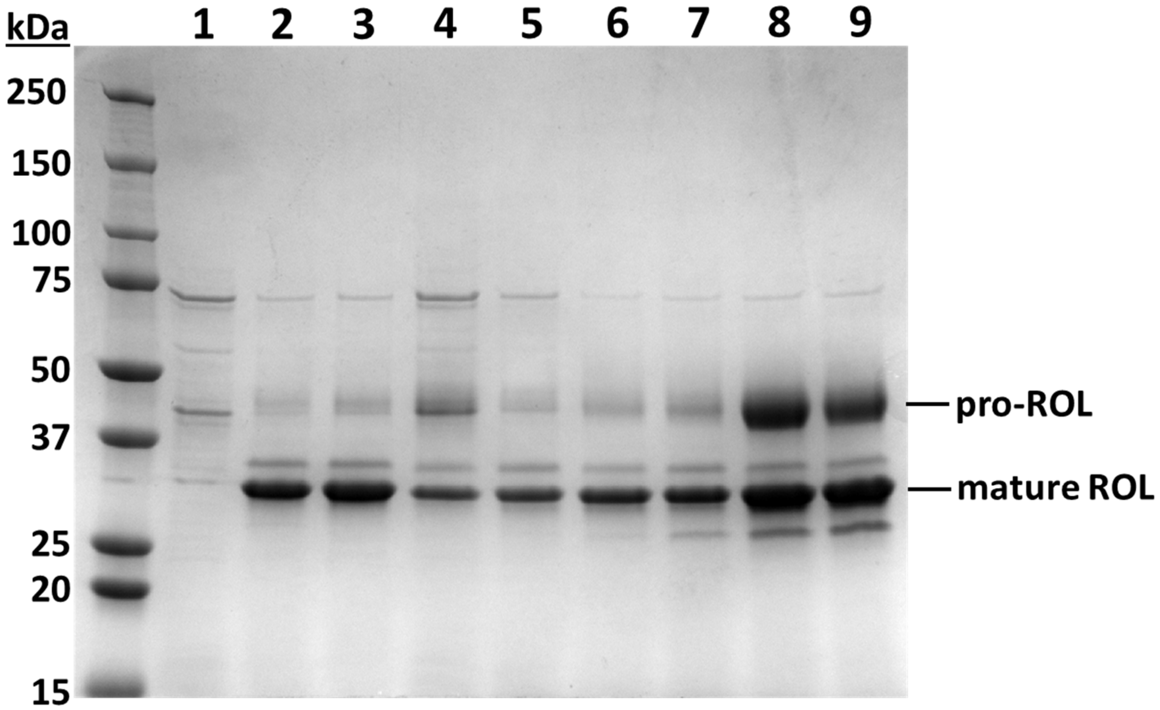

Figure S4. SDS-PAGE analysis of the ROL mutants expressed from *P.pastoris*. (1) Negative Control. (2,3) Wild-type ROL. (4,5) ROL_4x. (6,7) ROL_7x. (8,9) ROL_10x.

Table S1. Relative activity and fold improvement of the single mutants of ROL_4x measured at 60°C and 45°C.

Mutant	Relative activity (60°C/45°C)	Fold Improvement
ROL_4x	24%	1.0
ROL_4x_A8E	38%	1.6
ROL_4x_S31D	22%	0.9
ROL_4x_S114N	33%	1.4
ROL_4x_N134Y	52%	2.2
ROL_4x_T136D	43%	1.8
ROL_4x_P163K	34%	1.4
ROL_4x_F173Y	43%	1.8
ROL_4x_Y187N	30%	1.2
ROL_4x_G193K	30%	1.2
ROL_4x_Q197H	55%	2.3
ROL_4x_Q197Y	47%	1.9
ROL_4x_G228D	34%	1.4
ROL_4x_G228S	22%	0.9
ROL_4x_S267L	57%	2.4

Table S2. Relative activity and fold improvement of the single mutants of ROL_4x and ROL_7x measured at 65°C and 45°C.

Mutant	Relative activity (65°C/45°C)	Fold Improvement
ROL_4x	6%	1.0
ROL_4x_N134Y	16%	2.8
ROL_4x_T136D	3%	0.6
ROL_4x_F173Y	5%	0.9
ROL_4x_Q197H	9%	1.6
ROL_4x_Q197Y	3%	0.5
ROL_4x_S267L	13%	2.3
ROL_7x	45%	8.0

Table S3. Relative activity and fold improvement of the single mutants of ROL_7x measured at 67°C and 45°C.

Mutant	Relative activity (67°C/45°C)	Fold Improvement
ROL_7x	19%	1.0
ROL_7x_A7R	32%	1.7
ROL_7x_L113Y	26%	1.3
ROL_7x_Q150F	3%	0.2
ROL_7x_Q150H	4%	0.2
ROL_7x_G155A	15%	0.8
ROL_7x_S171F	16%	0.8
ROL_7x_V175F	81%	4.2
ROL_7x_G177C	125%	6.4
ROL_7x_T199V	24%	1.2
ROL_7x_F216Y	52%	2.7
ROL_7x_S223Y	4%	0.2
ROL_7x_F261Y	3%	0.2

Table S4. Relative activity and fold improvement of the single mutants of ROL_7x and ROL_10x measured at 70°C and 45°C.

Mutant	Relative activity (70°C/45°C)	Fold Improvement
ROL_7x	2%	1.0
ROL_7x_V175F	10%	4.5
ROL_7x_G177C	14%	6.1
ROL_7x_F216Y	3%	1.2
ROL_10x	121%	54.4
